# Challenges in the Diagnosis of Autoimmune Hepatitis in Patients With Chronic Viral Hepatitis C: A Case Series and Review of the Literature

**DOI:** 10.7759/cureus.96764

**Published:** 2025-11-13

**Authors:** Sayan Malakar, Sanjit Kumar, Rahul Jangra, Mayank Agarwal, Anubhav Parwar, Krishna P Kohli, Saurabh Mishra, Akriti Bhardwaj, Arvind Kumar, Ajay Singh, Dheeraj K Yadav, Abhishek Tiwari, Akhil Gandhi, Gaya Shukla, Sumit Rungta

**Affiliations:** 1 Gastroenterology, Sanjay Gandhi Postgraduate Institute of Medical Sciences, Lucknow, IND; 2 Gastroenterology, King George's Medical University, Lucknow, IND; 3 Medical Gastroenterology, King George's Medical University, Lucknow, IND; 4 Internal Medicine, King George's Medical University, Lucknow, IND; 5 Pathology, King George's Medical University, Lucknow, IND

**Keywords:** anti-liver kidney microsomal antibody (anti-lkm), anti-nuclear antibody, anti-smooth muscle antibodies, autoimmune cirrhosis, autoimmune hepatitis (aih), autoimmune hypothyroidism, chronic hepatitis c infection, chronic viral hepatitis, hepatitis c (hcv) infection, liver cirrhosis

## Abstract

Circulating non-organ-specific autoantibodies are commonly found in patients with chronic hepatitis C (CHC). Following the therapy for CHC, circulating autoantibodies such as anti-nuclear antibody (ANA), anti-smooth muscle antibody (ASMA), and anti-liver kidney-muscle-1 (LKM-1) antibodies tend to disappear; however, a subset of patients may develop overt immune-mediated disorders, including autoimmune hypothyroidism and autoimmune hepatitis (AIH). Development of AIH following CHC has been documented in the literature. Sometimes two conditions co-exist. Viral clearance unmask the features of overt AIH in such a group of patients. However, diagnosing AIH in the background of CHC and circulating autoantibodies is challenging. Data regarding the clinical implications of circulating autoantibodies and the risk of development of AIH in patients with CHC is scarce. Here, we present three cases of AIH that were diagnosed following successful treatment of CHC. The literature review aims to focus on the prevalence of circulating ANA, ASMA, and anti-LKM-1 in patients with CHC and their clinical implications.

## Introduction

Chronic hepatitis C (CHC) infection is associated with numerous immunopathological manifestations. Autoantibody production to overt immune-mediated diseases, such as immune-complex vasculitis, arthritis, Sjögren’s syndrome, mixed cryoglobulinemia, B-cell lymphoma, and autoimmune thyroid disorders, is associated with CHC [1-3]. Up to 50% of patients with CHC infection have been found to have anti-nuclear antibody (ANA), anti-smooth muscle antibody (SMA), anti-liver kidney microsomal type 1 (LKM1), anti-soluble liver antigen (SLA), anti-liver cytosol type 1 (LC1), and anti-mitochondrial antibody (AMA) [4-6]. Circulating autoantibodies in patients with CHC infection should be interpreted cautiously. Autoantibodies often persist following successful therapy of CHC infection; hence, the diagnosis of autoimmune hepatitis (AIH) should only be made based on typical liver histological findings. The development of anti-LKM1 antibodies after prolonged HCV infection indicates overt AIH type 2, occurring in 0-7% of CHC patients [7,8]. CYP2D6 is the main target of anti-LKM-1 in both AIH-2 and CHC [9,10]. There is limited information in the literature on the challenges and dilemmas of diagnosing and managing AIH in patients with CHC infection, particularly in the presence of circulating non-organ-specific antibodies (NOSA) [11,12]. This case series will focus on the challenges in the diagnosis of AIH in patients with a history of CHC infection, with a brief literature overview of the prevalence of circulating NOSA in such patients.

## Case presentation

Case 1

**Figure 1:**
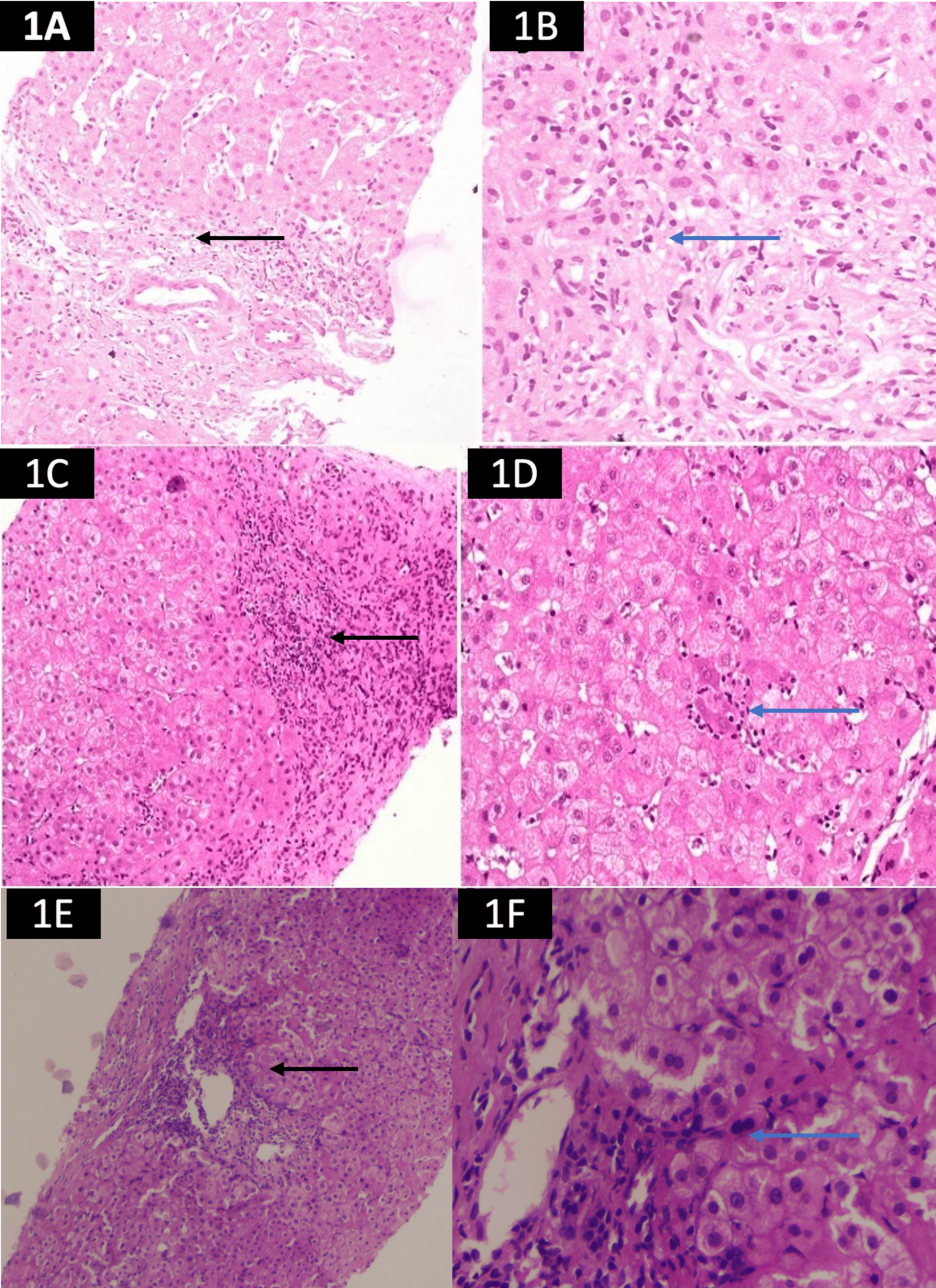
Liver biopsies showing the features of autoimmue liver diseases in patients following the elimination of hepatitic C virus infection 1A: Histology of the liver biopsy shows features of acute hepatitis with moderate interface hepatitis (A: black arrow; 200x haematoxylin and eosin). 1B: The portal tracts show moderate lymphoplasmacytic infiltration (blue arrow) (400x and H&E). 1C: Hepatocyte shows moderate interface hepatitis (C black arrow) (200x and H&E). 1D: Occasional emperipolesis (blue arrow) suggestive of AIH (400x and H&E). 1E: Severe interface hepatitis with occasional lymphoplasmacytic infiltration (200x and H&E). 1F: Severe interface hepatitis (400x and H&E).

A 57-year-old female presented with generalised body pain and a rise in aspartate aminotransferase (AST) and alanine aminotransferase (ALT). She was diagnosed with autoimmune hypothyroidism three years ago and was on 75 micrograms a day of thyroxine supplement. On further evaluation, the patient’s anti-hepatitis C virus antibody came out to be positive. Her HCV ribonucleic acid (RNA) was 5.7 x 10^4^/mm^3^. Ultrasound and transient elastography (TE) showed no evidence of advanced fibrosis or decompensation (Table 1). The patient was treated with sofosbuvir and daclatasvir for three months, and she achieved a sustained virological response (SVR) following direct-acting anti-viral (DAA) therapy. However, her liver function test remained elevated (AST: 97 U/L; ALT: 66 U/L; alkaline phosphatase (ALP): 178 U/L) after six months of achieving SVR. On further evaluation, the patient’s ANA and ASMA came out to be positive (Table 2). Serum immunoglobulin-G was elevated to 2,720 g/dL. She underwent a percutaneous liver biopsy, which revealed features of AIH (Figure 1A). Her simplified AIH score was 7, and she was started on steroids (Table 2). Following the steroid (prednisolone: 40 mg per day), the patient’s LFT and IgG improved. Subsequently, azathioprine was added, and prednisolone was tapered to 5 mg/day.

**Table 1:** Laboratory parameters of the patient presenting with hepatitis C infection Abbreviation: AST: aspartate aminotransferase; ALT: alanine aminotransferase; ALP: alkaline phosphatase; INR: international normalised ratio; LSM: liver stiffness measurement; HCV: hepatitis C virus; RNA: ribonucleic acid

Parameters (Normal value)	Patient 1	Patient 2	Patient 3
Age (in years)	57	46	67
Gender	Female	Female	Female
HCV RNA (non-detectable)	5.7 x 10^4^/mm^3^	7.6 × 10^5^/mm^3^	4.5 x 10^3^/mm^3^
LSM (kPa)	7.2	5.4	Not available
Treatment	Sofosbuvir and daclatasvir	Sofosbuvir and daclatasvir	Sofosbuvir and daclatasvir
Hemoglobin (g/dL) (11-15 g/dL)	11.2	9.4	7.2
Total leukocyte count (per/mm^3^) (4000-11,000 per/mm^3^)	4500	1.78	3100
Platelet count (lacs/mm^3^) (1.5-4.5 lacs/mm^3^)	0.97	1.3	0.40
Bilirubin mg/ dL (<2 mg/dL)	2.1	0.6	4
AST (U/L) (<40 U/L)	112	78	184
ALT(U/L) (<40 U/L)	76	44	86
ALP (U/L) (<150 U/L)	200	189	280
Albumin (g/dL) (3.5-4.5 g/dL)	3.1	3.8	4.2
Protein (g/dL) (6.5-8 g/dL)	6.3	6.8	7.1
INR	1.3	1.4	1.8

**Table 2:** Parameters following sustained virological response Abbreviation: AST: aspartate aminotransferase; ALT: alanine aminotransferase; ALP: alkaline phosphatase; AIH: autoimmune hepatitis; AMA: anti-mitochondrial antibody; AMA: anti-mitochondrial antibody; ANA: antinuclear antibody; ASMA: anti-smooth muscle cell antibody; IgG: immunoglobulin-G; LKM: liver-kidney-muscle; LSM: liver stiffness measurement; HCV: hepatitis C virus; RNA: ribonucleic acid

Parameters	Patient 1	Patient 2	Patient 3
Bilirubin mg/ dL (<2 mg/dL)	2.1	4.1	7.6
AST (U/L) (<40 U/L)	97	516	1480
ALT(U/L) (<40 U/L)	66	386	1150
ALP (U/L) (<150 U/L)	178	219	345
Albumin (g/dL) (3.5-4.5 g/dL)	3.5	3.2	2.7
Protein (g/dl) (6.5-8 g/dL)	7.1	6.1	5.2
INR (<1.5)	1.3	1.4	1.9
ANA	Positive (1:80)	Positive (1:80)	Positive (1:80)
ASMA	Positive (1:100)	Positive (1:80)	Negative
IgG (g/dL) (<1800 mg/dL)	2720	3300	1980
AMA	Negative	Negative	Negative
Other autoimmune diseases	Autoimmune hypothyroidism	Autoimmune hypothyroidism	Autoimmune hemolytic anemia
Liver biopsy	Moderate interface hepatitis and lymphoplasmacytic infiltration	Moderate interface hepatitis and emperipolesis	Severe interface hepatitis and lymphoplasmacytic infiltration
Repeat HCV RNA	Negative	Negative	Negative
Simplified AIH score	8	8	7
Revised AIH score	26	25	20

Case 2

A 46-year-old female, known case of hypothyroidism, presented with a long-standing history of pain in multiple joints and elevated liver enzymes. She was screened for rheumatoid arthritis and other connective tissue disorders, which were negative. In view of elevated liver enzymes, the patient was referred to us. She was diagnosed with CHC (HCV RNA: 7.6 × 10^5^) without any evidence of advanced fibrosis (Table 1). She achieved SVR after receiving sofosbuvir and daclatasvir for three months. However, her polyarthralgia persisted, and repeated investigation demonstrated raised bilirubin (4.1 mg/ dL), aspartate aminotransferase (AST: 516 U/L), and alanine transaminase (ALT: 386 U/L). Hence, she was evaluated for other etiologies of chronic hepatitis. Her ANA and ASMA were positive, and IgG (3,300 g/dL) was elevated (Table 2). HCV RNA was repeated, and it was negative. Liver biopsy showed evidence of AIH (Figures 1C-1D). She was successfully treated with steroids and azathioprine. 

Case 3

A 67-year-old female presented with a history of recurrent jaundice for the last two years. The history of jaundice was not associated with any pruritus or liver-related decompensation. On further inquiry, the patient revealed that she had taken treatment with sofosbuvir and daclatasvir for CHC five years ago. After three months of therapy, she achieved SVR. In between, she was completely asymptomatic. Her current investigations revealed direct hyperbilirubinemia and high liver enzymes (bilirubin: 7.6 mg/dL; AST: 1,480 U/L; ALT: 1,150 U/L; ALP: 345 U/L) (Table 2). Budd-Chiari syndrome and Wilson disease were ruled out, and there was no history of any recent drug intake. Her peripheral smear revealed evidence of hemolysis. On further evaluation, her lactate dehydrogenase was elevated (1,578 U/L) with a low haptoglobin, suggestive of hemolytic anemia. As her coomb test was positive, she was diagnosed with autoimmune hemolytic anaemia. The current hepatitis infection panel was negative (HCV RNA, hepatitis B deoxyribonucleic acid (HBV DNA), IgM anti-HAV, IgM anti-HEV, and IgM anti-HBc). Her autoimmune profile came back positive (ANA: 1:100 positive; ASMA: 1:80 positive; IgG: 1880 g/dL). Ultrasound of the abdomen revealed coarse echotexture of the liver; however, the margins were regular. There was no evidence of portal hypertension. She underwent transjugular liver biopsy, which revealed marked interface hepatitis compatible with acute severe AIH (Figures 1E-1F).

The patient was started on steroids; however, the patient did not improve and developed hepatic encephalopathy. Liver transplant work-up was initiated promptly; however, the patient died of multiorgan failure and sepsis after nine days of hospitalization.

## Discussion

The cases illustrated the dynamic interaction between liver-directed autoimmunity and HCV infection. Even after an SVR, patients presented with AIH. Circulating autoantibodies are common in patients with HCV infection; however, persistently elevated liver enzymes following SVR were the clue to the diagnosis of a concurrent or alternative cause of liver injury. Cirrhotic patients with concurrent AIH and HCV may benefit greatly from viral clearance, and DAA-induced viral clearance may be linked to the disappearance of severe immune-mediated diseases. There is also evidence of treatment-related emergence of AIH after DAA-induced SVR and possible worsening of underlying AIH and autoimmune phenomena after successful DAA treatment of HCV [13-17]. In case reports by Covini et al. and Matsumoto et al., the patient presented with elevated liver enzymes during DAA treatment and was diagnosed to have AIH, which improved after steroid treatment [16,17]. Covini et al. reported a case of CHC in a 72-year-old lady who developed acute hepatitis after two weeks of therapy with DAA. On liver biopsy, she showed frank features of AIH. She was managed with immunosuppressive therapy [16]. Serological proof of this ongoing autoimmune phenomenon was revealed by the Swiss Hepatitis-C Cohort research, a prospective cohort study [18]. Notably, they discovered that most patients remained autoantibody-positive even when DAA-induced SVR. More surprisingly, at least one autoantibody developed after SVR in 27% of patients who were seronegative before therapy, with ANA and SMA appearing de novo in 16% and 8% of patients, respectively [18]. This emphasises that not everyone experiences immunologic normalisation following viral clearance. The exact mechanism of autoimmunity triggering AIH in patients with HCV infection is unknown. The successful removal of the viral trigger upset an immunological tolerance state, which subsequently caused a flare-up of clinically obscured AIH. The significant influence of HCV on B-cell biology is recognised as the pathophysiological mechanism for the persistence and de novo emergence of autoimmunity following SVR [18]. There are several ways the virus interacts with B cells: C3d complement fragment, which coats the HCV particle, binds to the C3d receptor, the CD-21 molecule, and the HCV envelope glycoprotein E2 binds to the transmembrane tetraspanin CD-817. These interactions promote the generation of autoantibodies and cryoglobulins by dramatically lowering the threshold for B-cell activation [19,20]. The B-cell clones that cause these symptoms have been shown to endure for a considerable amount of time beyond a DAA-induced recovery, and they are occasionally linked to late relapse vasculitis, suggesting that the immunologic dysregulation outlasts the virus [21-23]. The pathogenesis of AIH following hepatotropic virus infection is not well understood. Hepatocyte damage, molecular mimicry, and antibodies against shared epitope have been implicated in the development of post-infection AIH [18-22].

As demonstrated by our third case, post-SVR AIH development can be severe and even fulminant. Thus, autoantibodies should be regarded as a possible risk factor for current or future liver damage, whether they are pre-existing or develop de novo during SVR. Patients should have long-term biochemical and clinical monitoring if they show ongoing autoantibody positivity or if they acquire new autoimmune serology after viral clearance. This is critical for ensuring early detection and intervention for autoimmune phenomena. Autoantibody testing during a chronic HCV infection is not recommended by current guidelines, either before or following DAA treatment [24,25]. Additional large-scale, long-term outcome studies are required to firmly correlate these serological findings with clinical endpoints and establish evidence-based follow-up strategies for this specific patient population.

Review of the literature

Prevalence of ANA, ASMA, and Anti-LKM1 Autoantibody in Patients With HCV Infection and Their Clinical Implications

Overall prevalence of NOSA in patients with HCV infection ranges from 20% to 50% [4,26-30]. Most commonly found autoantibodies are ASMA, followed by ANA [26-32]. In a study consisting of 308 patients with CHC, ASMA and ANA were present in 17.8% and 6%, respectively [29]. Another study by Stroffolini et al. included more than 500 patients with CHC. The overall prevalence of ANA and ASMA was 27.3% and 20%, respectively [30] (Table 3). The predictors of autoantibody positivity among patients with HCV infections are higher IgG, female gender, and older age. Stroffolini et al. found that a higher IgG level is an independent risk factor for developing autoantibodies, whereas Cassani et al. found older age and female sex as the risk factors for having autoantibodies [4] (Table 3). Data regarding the clinical implications of these circulating autoantibodies have been controversial, with different results in different studies. Amin et al. and Cassani et al. have shown that circulating autoantibodies are associated with elevated liver enzymes and more severe disease; however, they do not interfere with the treatment response [4]. Anti-LKM1, anti-LC1, and anti-SLA are rare in this group of patients [18,28-30]. The impact of treatment on the circulating autoantibodies was described in a study by Terziroli Beretta-Piccoli et al. [18]. Following treatment in 235 patients, ANA and ASMA disappeared in 34% and 52% patients, respectively [18]. Interestingly, on follow-up, one or more autoantibodies appeared in 27% pre-treatment-negative patients. AIH is a rare cause of chronic liver disease and is often associated with autoimmune extrahepatic manifestations, such as autoimmune hypothyroidism [33-35]. However, autoimmune hypothyroidism is also associated with CHC infection and interferon therapy, posing more challenges to the physicians [36,37]. Two of our patients had autoimmune hypothyroidism, corresponding to previous reports. There are only a few case reports regarding the development of AIH during DAA therapy in patients with CHC, as described above [16,17]. Numerous reports are available on the infections triggering autoimmune hepatitis and other autoimmune diseases [38-40]. Both viral and bacterial infections are known to trigger autoimmunity [39-42]. The association between CHC and AIH needs more attention.

**Table 3:** Prevalence of AIH-related autoantibodies in chronic hepatitis C infections Abbreviations: AIH: autoimmune hepatitis; ANA: antinuclear antibody; ASMA: anti-smooth muscle cell antibody; HCV: hepatitis C virus; IgG: immunoglobulin G; LKM: liver-kidney-muscle

Authors	No. of patients	Prevalence of autoantibodies	Clinical implications
Terziroli Beretta-Piccoli et al. [18]	235	One autoantibody before treatment was found in 175 (74%) patients; 32 (14%) were positive for 2 autoantibodies; no patient was positive for anti-SLA, anti-LC1, or typical AMA	ANA disappeared in 34%, ASMA in 52% and anti-LKM1 in one of two patients after successful treatment, but, unexpectedly, one or more autoantibodies appeared in 27% of pre-treatment negative subjects
Amin et al. [26]	50	Antinuclear antibody, SMA, and LKM-1 Ab were also present in 36% of CHC patients and were related to disease severity	Autoantibody-positive patients with hepatitis C have higher liver enzymes and severe disease
Daschakraborty et al. [27]	35	Any antibody: 43%; ANA: 20%; ASMA: 26%	Detection of autoantibodies is frequent in patients with chronic liver disease and HCV
Muratori et al. [28]	348	ANA: 6%; ASMA: 17.8%; Anti LKM1: 8%	Patients receiving IFN therapy for HCV infection seropositive for anti-LKM1 are more susceptible to developing autoimmune thyroid disease
Rigopoulou et al. [29]	138	ANA: 3.6%; ASMA: 4.3%; Anti LKM1: 13%	Anti-LC1 and anti-SLA antibodies are not seen in anti-LKM1-negative HCV patients
Stroffolini et al. [30]	502	ANA: 20%; ASMA: 27.3%; Anti-LKM1: 2.2%	IgG > 2 g/ dL is associated with NOSA. Presence of NOSA does not affect the outcome following treatment for HCV
Peng et al. [31]	48	ANA: 23%; ASMA: 78%	The speckled pattern of ANA is the most common among patients with HCV infection. ANA-positive patients with HCV infections have higher liver enzymes
Cassani et al. [4]	290	ANA: 9%; ASMA: 20%; Anti-LKM1: 6%	Autoantibodies are more prevalent in female patients, and they have higher biochemical and histological activity
Squadrito et al. [32]	283	ANA: 7.7%; ASMA: 12.7%; Anti-LKM1: 0.7%	The presence of cirrhosis and older age are the risk factors for NOSA in patients with HCV infection

Longitudinal long-term follow-up data on the development of AIH in autoantibody-positive patients with HCV infection are lacking. The presence of autoantibodies is associated with elevated liver enzymes in patients with HCV infection; however, liver biopsy should be done to rule out AIH in these patients.

## Conclusions

HCV infection may trigger AIH. The circulating autoantibodies, such as ANA and ASMA, are present in 40-50% of patients with HCV infection. In case of persistent autoantibodies and elevated liver enzymes following SVR, a liver biopsy may help establish the diagnosis of AIH.
